# Enhancing Temperature Sensitivity of the Fabry–Perot Interferometer Sensor with Optimization of the Coating Thickness of Polystyrene

**DOI:** 10.3390/s20030794

**Published:** 2020-01-31

**Authors:** Tejaswi Tanaji Salunkhe, Dong Jun Lee, Ho Kyung Lee, Hyung Wook Choi, Sang Joon Park, Il Tae Kim

**Affiliations:** 1 Department of Chemical and Biological Engineering, Gachon University, Seongnam-si, Gyeonggi-do 13120, Korea; tejaswisalunkhe235@gmail.com (T.T.S.); passeddays@naver.com (D.J.L.); ghrud0722@gmail.com (H.K.L.); 2 Department of Electrical Engineering, Gachon University, Seongnam-si, Gyeonggi-do 13120, Korea; chw@gachon.ac.kr

**Keywords:** Fresnel reflection, polystyrene, single-mode fiber, temperature sensitivity

## Abstract

The exploration of novel polymers for temperature sensing with high sensitivity has attracted tremendous research interest. Hence, we report a polystyrene-coated optical fiber temperature sensor with high sensitivity. To enhance the temperature sensitivity, flat, thin, smooth, and air bubble-free polystyrene was coated on the edge surface of a single-mode optical fiber, where the coating thickness was varied based on the solution concentration. Three thicknesses of the polystyrene layer were obtained as 2.0, 4.1, and 8.0 μm. The temperature sensor with 2.0 μm thick polystyrene exhibited the highest temperature sensitivity of 439.89 pm °C^−1^ in the temperature range of 25–100 °C. This could be attributed to the very uniform and thin coating of polystyrene, along with the reasonable coefficient of thermal expansion and thermo-optic coefficient of polystyrene. Overall, the experimental results proved the effectiveness of the proposed polystyrene-coated temperature sensor for accurate temperature measurement.

## 1. Introduction

Temperature is a significant parameter in various fields, including superconducting magnets, chemistry, biotechnology, aerospace, the energy sector, and so on. Traditional temperature measurement tools are mainly based on electronic devices and thermocouples. However, electronic devices cannot be utilized in rigorous environments, such as in the existence of corrosive substances, at extremely low or high temperature, and in chemical environments. In this regard, substantial attention has been paid to temperature sensor research because of the vigorous usage of such sensors in the aforementioned fields over the last few decades [1,2].

Most importantly, among the different types of temperature sensors, optical fiber temperature sensors have successfully replaced traditional sensors owing to their distinctive advantages, including stability, immunity to electromagnetic interference, reusability, durability against stringent environments, high sensitivity, wide dynamic range, multiplexing capability, and fast response in a non-electrical operation [3,4]. A variety of optical fibers have been utilized in the field of optical fiber temperature sensors such as fiber Bragg gratings (FBGs) [2,4,5,6], long period gratings (LPGs) [7,8,9], hollow core fibers (HCF) [10,11], multimode interference-based optical fibers (MMF) [12], and micro-bend fibers coupled with thin films [13,14]. Despite their many advantages, these optical fiber temperature sensors have some shortcomings, including cross-sensitivity, poor repeatability, and complexity. Meanwhile, Fabry–Perot interferometer sensors could be based on the Fresnel reflection technique for temperature measurement, which have some advantages including ease of preparation, suitable for real time operation, good repeatability, and high sensitivity [3,14]. Fabry–Perot interferometer optical fiber sensors demonstrate an interference phenomenon resulting from differences in the refractive index (RI) of the three different materials (optical fiber, coating material, and air) [15,16,17]. For example, Fabry–Perot interferometer sensors were developed by coating the optical fiber with agarose [18,19], polymers [20,21,22,23], other porous silica xerogels [24], carbon nanotubes [25], and ZnO [11]. In previous studies, HCFs, MMFs, single mode fibers (SMFs), photonic crystal fibers (PCFs), and plastic optical fibers (POFs) [10,11,21,26,27] have been utilized for humidity and temperature measurements. The sensing mechanism is generally based on small variations of the RI due to the temperature change with respect to the thicknesses of the thermosensitive coating material.

Utilization of polymers with higher coefficients of thermal expansion (CTE) and thermo-optic coefficients (TOC) than that of silica has been researched. Various research on the temperature sensors with thermosensitive polymers has been conducted as well. For instance, Rong et al. explored the polyvinyl alcohol (PVA) polymer for the application to the Fabry–Perot interferometer sensor [21], Romano et al. developed a polydimethylsiloxane (PDMS) coated microfiber mode interferometer sensor [23], Salunkhe et al. demonstrated the Fabry–Perot interferometer sensor with polycarbonate (PC) and poly (methyl methacrylate) (PMMA) [28], EI-Amassi et al. applied the polystyrene (PS) for the temperature sensor with a photonic crystal fiber [29], and Esposito et al. explored the PS coated LPG for temperature sensing [30]. It is acknowledged that the change of the thermal optic coefficient is the prime motive for the temperature-derived index change in the polymer, which plays a crucial role in determining the device performance; thus achieving this feature has been a targeted research goal for optical fiber temperature sensors. The TOC and CTE are responsible for the wavelength shift, RI and volume change of polymer with temperature. Consequently, the associated wavelength shift is very sensitive to small temperature changes [21,22,23]. To the best of our knowledge, no studies have been reported for the PS coated Fabry–Perot interferometer sensor with a single mode fiber, where the sensitivity was changed by varying the thickness of PS coating. Considering these requirements, we are exploring PS with a TOC of − 1.2 × 10^−4^ °C^−1^ and CTE of 2.2 × 10^−4^ °C^−1^ for Fabry–Perot interferometer temperature sensor application [29,31].

In this study, we explored a thermosensitive polystyrene (PS) coated Fabry–Perot interferometer temperature sensor, which can demonstrate an unsophisticated temperature sensing technique with high sensitivity. A PS coated Fabry–Perot interferometer sensor is developed on the principle of Fresnel reflection. To improve the temperature sensitivity, the surface coating thickness of the PS is tuned by simply changing the concentration of the PS solution. The PS-coated temperature sensor shows a red shift of the wavelength with increasing temperature, whereas the wavelength is blue-shifted with decreasing temperature. The developed temperature sensor exhibits temperature sensitivity variations based on the coating thickness. The as-prepared sensor with a thickness of 2.0 μm of PS demonstrated the highest temperature sensitivity of 439.89 pm °C^−1^ in the temperature range of 25–100 °C. Our sensor would exhibit wide potential applications, in the field of chemical, biochemical, physics, biological or in other areas wherever high sensitivity is required.

## 2. Sensing Principle

### 2.1. Fresnel Reflection Principle

**Figure 1:**
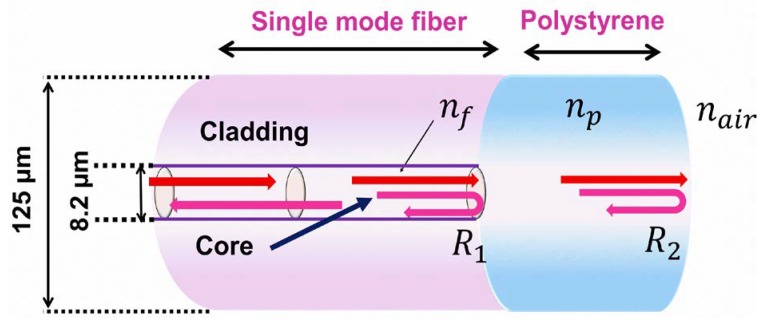
Schematic diagram of the proposed polystyrene-coated temperature sensor.

Figure 1 demonstrates the basic configuration of the Fabry–Perot interferometer temperature sensor based on the Fresnel reflection principle. Fresnel reflection is the basic optical phenomenon that occurs at the interface between media with different RI. The two reflection surfaces are on the sensing head, i.e., fiber–polymer and polymer−air, where R_1_ and R_2_ are the Fresnel reflection coefficients for two surfaces, respectively. The reflection of light happened on the both surfaces is the Fresnel reflection; consequently, R_1_ and R_2_ can be determined as follows [32,33,34].

(1) $$R_{1}=\frac{{\left(n_{f}-n_{p}\right)}^{2}}{{\left(n_{f}+n_{p}\right)}^{2}}\;\;\;\;\;\;\;\;\;\;$$

(2) $$R_{2}=\frac{{\left(n_{p}-n_{air}\right)}^{2}}{{\left(n_{p}+n_{air}\right)}^{2}}\;\;\;\;\;\;\;\;\;$$

where, $n_{f}$,$\;n_{p}$, and $n_{air}$ are the RI of the fiber, PS, and air, respectively. When the two reflected light beams back into SMF, they interfere with each other due to their different optical paths. The two reflected light intensities backed into the SMF are denoted as $r_{1}$ and $r_{2}$ respectively. Thus performance of the cavity can be approximated by a two beam interferometric model, and the output intensity of r can be expressed as [33,35].

(3) $$r=r_{1}+\;r_{2}\pm 2\sqrt{r_{1}}r_{2}Cos\;\left(\emptyset \right)\;\;\;\;\;\left(\left(-\right)\;\;sign\;at\;n_{air}\leq n_{p}\right)\;\;\;\;\;\;\;\;\;\;\;\;\;\;\;\;\;\;$$

where reflection intensity of the interference signal is r, and optical phase difference is denoted as ∅, which can be written as.

(4) $$\emptyset =\frac{4\pi n_{p}L}{\lambda }\;\;\;\;\;\;\;$$

where, n_p_ is the RI of PS; λ is the wavelength of light source. Optical path (n_p_L) is responsible for the phase difference between fiber–polymer and polymer–air. The thickness of the PS coating is expressed as L. Free spectral range (FSR) represents the periodicity of the spectral fringes of the spectra [33].

(5) $$\mathrm{FSR}=\;\frac{\lambda_{m}\lambda_{m+1}}{2n_{p}L}\;\;\;$$

where m is an integer, $\lambda_{m}$ is the wavelength of the m^th^ peak in the spectra, and $\lambda_{m+1}$ is the wavelength of the spectra next to the m^th^ peak. Variations in the cavity length caused by temperature change generate phase shifts in the interference signal that can be retrieved by tracking the wavelength shift of the interference spectrum using an optical spectrum analyzer (OSA). Consequently, the m^th^ wavelength (λ_m_) of the spectrum minimum satisfying the condition ∅=2mπ is expressed by Equation (6) [21]

(6) $$\lambda_{m}=\;\frac{2n_{p}L}{m}\;\;\;\;\;\;\;\;\;$$

According to Equation (6), the thickness of the PS coating and the refractive index of the PS are responsible for the resonant dip. The thickness and RI of PS are functions of the temperature. When ambient temperature changes, wavelength ($∆\lambda_{m}$) of the resonant peak will be given by:

(7) $$∆\lambda_{m}=\frac{2}{m}\;\left(\;\frac{dn_{p}}{\mathrm{dT}}L+n_{p}\frac{\mathrm{dL}}{\mathrm{dT}}\;\right)∆T\;\;\;\;\;\;\;\;$$

Equation (7) demonstrates that the temperature variations affect the two parameters: one is the RI of the PS film changes ($\frac{dn_{p}}{\mathrm{dT}}$) and the other is temperature induced length variation ($\frac{\mathrm{dL}}{\mathrm{dT}}$). Hence, the polymer properties are responsible for the temperature–induced shift of a particular peak. The thermo-optical parameter and CTE of PS are − 1.2 × 10^−4^ °C^−1^ and 2.2 × 10^−4^ °C^−1^, respectively (refer to Table 1).

### 2.2. Sensor Fabrication 

A schematic diagram of the Fabry–Perot interferometer sensor is presented in Figure 1, where the fiber is a standard single mode fiber-28 (SMF-28; New York, United States) with cladding and a core diameter of 125 and 8.2 μm, respectively. The SMF tip is not flat at the edge because the fiber cladding is covered with very thin coating consisting of the zirconium oxide. It is developed with a face angle, which could decrease return loss as well as create uniform coating of the polymer. For this work, polystyrene (PS) was selected as the coating material as it can form a good, clear, colorless, viscous solution at a low wt.%. Since the adhesive force between SMF-28 and PS is strong, PS can form a uniform coating in the range of 5−20 wt.% PS in tetrahydrofuran (THF). This superior characteristic is very important for forming high-quality sensors [21,22,23].

The glass transition temperature of PS was reported as 100 °C, and the nominal RI of PS is 1.59 at room temperature [29,32]. The sensing head with the PS coating was prepared by using the simple dip coating method. In brief, the calculated amount of PS (Mw = 350000, Sigma–Aldrich) granules was weighed and mixed into the weighed amount of THF (Sigma–Aldrich, 99.9%) under stirring at 50 °C for 1 h to allow full dissolution of the PS granules and formation of a homogeneous PS solution (5−20 wt.%). Thereafter, the tip of the bare SMF (without polymer coating) was cleaned with isopropanol for 10 mins and allowed to dry at 25 °C. A tip of the SMF was dipped into the PS solution for 10 mins to obtain a flat and air bubble-free coating with controlled thickness by utilizing solutions with different concentrations. After that, the SMF was kept in a vacuum oven at 65 °C for 15 mins for solidification. The same process was repeated five times to obtain durable sensor with stable coating. Finally, the SMF sample with five coatings was dried for 1 h in vacuum oven at 65 °C. The obtained coating thickness of PS was 2.0, 4.1, and 8.0 μm with the use of 5, 15, and 20 wt.% PS solution, respectively.

### 2.3. Measurement Setup

**Figure 2:**
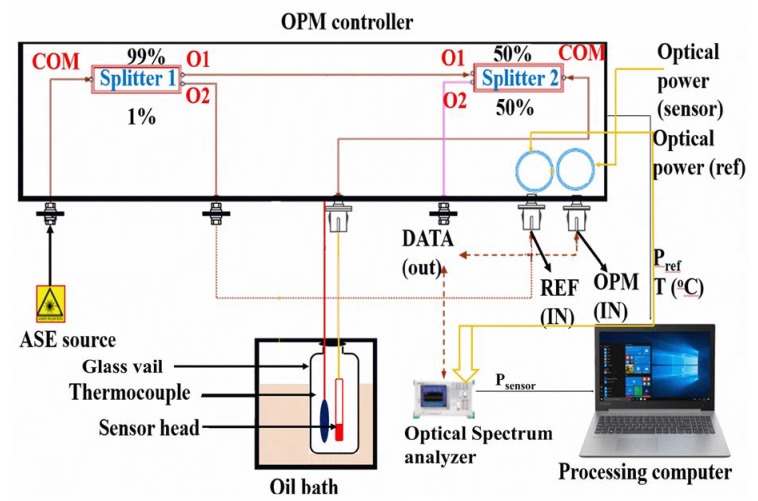
Experimental setup for the temperature sensing measurement.

The setup for the temperature sensitivity measurement is illustrated in Figure 2; the system analyzes the wavelength shift with respect to the temperature change of the thermosensitive polymer-coated extrinsic Fabry–Perot interferometer sensor based on the Fresnel reflection technique. The measurement system comprises a C-band amplified spontaneous emission (ASE) broadband light source with a wavelength range of 15,351,570 nm and central wavelength of 1550 nm, which is the wavelength used in telecommunications. The other components include an optical power monitoring (OPM) controller system, optical spectral analyzer (OSA; model MS9710C, Anritsu, Kanagawa Prefecture, Japan) with the minimum resolution of 0.05 nm, oil bath, PS-coated temperature sensor, glass vial, and processing computer. The ASE light source supplies light to the OPM, which is coupled twice by the optical fiber coupler; first, it is coupled into coupler (I) and divided into two parts 1%:99%. Thus, 1% of the light from the ASE is supplied to the optical power monitoring reference fiber, and the other end of this reference fiber is connected to the OSA to provide a reference signal to the OSA. The remaining 99% of the light is provided to directional coupler (II), 50% of the power from coupler (I) is delivered to the fabricated sensor, and OSA receives 50% of the power reflected from the sensor.

### 2.4. Temperature Response Test

For the temperature sensitivity measurement, the PS coated Fabry–Perot interferometer sensor head with the thermocouple (Giltron GT 307/08, New Taipei, Taiwan) having a resolution of ± 0.1 °C was inserted into the glass vial, and the vial was dipped into the oil bath to control temperature. Light triggered from the C-band amplified spontaneous emission broadband light source was inserted into the sensor area through POM controller. The temperature was recorded with a thermocouple while ramping it from 25 to 100 °C in 5 °C intervals. Thereafter, the system was allowed to cool naturally from 100 to 25 °C. The spectral response at different temperatures was recorded by the OSA at 1 nm resolution. To obtain credible data, including the spectral response, the system was maintained at the constant temperature for at least 5 mins. The same experiment was done five times of every sensor for checking the stability. The average sensitivity data was taken for temperature sensitivity.

## 3. Results and Discussion 

The principal criteria of the effective Fabry–Perot interferometer sensors is the RI of the coating material should be greater than the fiber and air [22]. Notably, the RI of the PS was higher than that of the silica (RI = 1.456) fiber and air (RI = 1), and the RIs of some reported polymer are summarized in Table 1 [36,37]. The PS coated sensor had the ability to produce the higher Fresnel reflection compared to (PC), poly (vinyl chloride) (PVC), Norland Optic adhesive-61 (NOA-61), and Norland Optic adhesive-65 (NOA-65). According to the Equation (3), r_2_ is higher than r_1_, which causes considerable fringe visibility. The large RI of PS could create phase differences for high Fresnel reflection. These RI differences between PS and optic fiber/air are responsible to the desired fringe visibility of the wavelength spectra, where the higher the difference of RI, the higher the fringe visibility [33]. In addition, adhesive properties of the polymer with fiber are another important characteristics of the polymer for the formation of a high quality sensor. PS shows good adhesive force with fiber in our system. RI and density change with respect to temperature are the common phenomenon of the thermosensitive polymer. These values are influenced by the sensitivity of the temperature sensor [33]. Moreover, PS exhibited much higher TOC than those of PVC, PC, NOA 61, NOA 65, and epoxy. To date, PMMA is the most widely utilized polymer for the application to the temperature sensor due to its high sensitivity and good adhesive characteristics [28]. However, it cannot be applicable for long temperature range of measurement. For instance, PMMA can be used up to the 90 °C at ambient conditions [4,5]. On the other hand, the PC coated temperature sensor could be operated up to 145 °C; however, it shows low sensitivity [28]. By considering all the facts, PS polymer would have been a more appropriate choice for the Fabry–Perot interferometer temperature sensor. 

**Figure 3:**
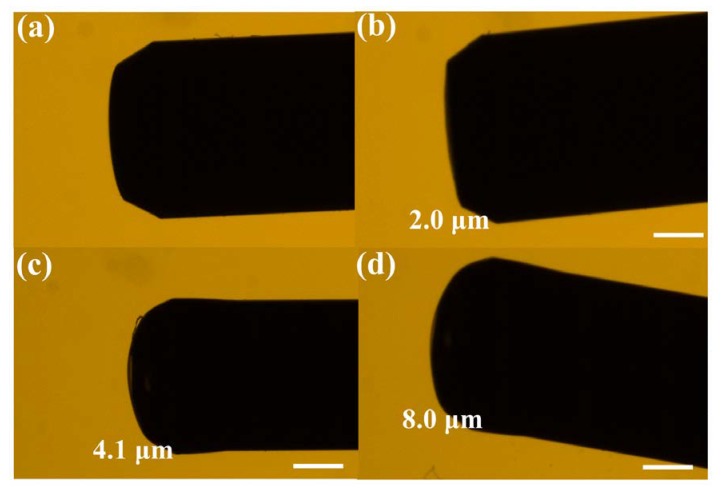
Optical microscopic images of SMF (**a**) without coating, and with PS coating; the coating thickness is (**b**) 2.0 μm, (**c**) 4.1 μm, and (**d**) 8.0 μm, scale bar corresponds to 35 μm.

**Figure 4:**
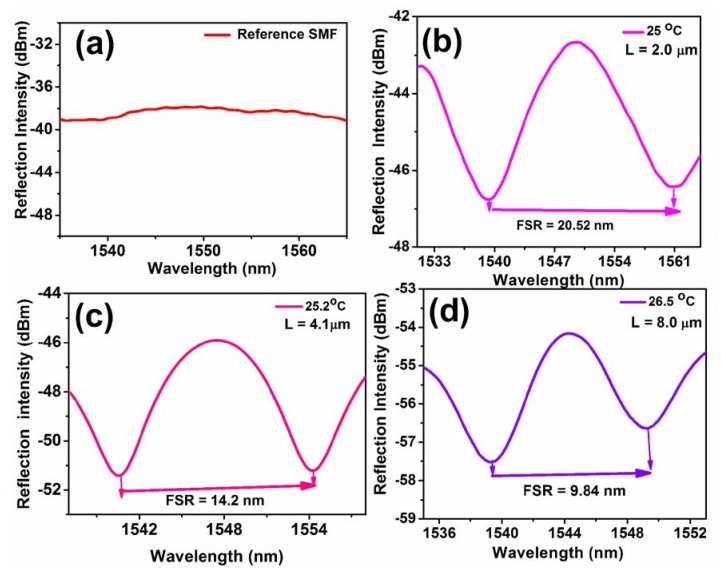
Reflection spectra of the proposed sensors with a different PS coating thickness: (**a**) bare SMF, (**b**) 2.0 μm, (**c**) 4.1 μm, and (**d**) 8.0 μm.

The main advantage of the as-prepared PS-coated Fabry–Perot interferometer sensor is the facile control of the thickness of PS by simply changing the concentration of the solution. This controlled thickness of the coating was confirmed by the optical microscopic images (Nikon Eclipse 80i Upright Fluorescent Microscope-6980), as shown in Figure 3. Figure 3a shows the surface end of the bare SMF, and Figure 3b–d shows the PS-coated Fabry–Perot interferometer with various thicknesses of 2.0, 4.1, and 8.0 μm, respectively. The images clearly show that the PS coating is uniform, thin, and bubble free. The spectral response of the bare SMF and PS-coated SMF sensors were measured, as shown in Figure 4. The reflection spectrum of bare SMF appeared as a straight line (Figure 4a), illustrating that most of the light was transmitted from the end of the SMF. Figure 4b−d show that the coating thickness affects the spectral response. The spectral responses indicate that a thicker coating produces dense interference patterns, whereas thinner coatings produce broad interference patterns [21,33].

However, several factors influence the interference patterns of the devices, such as the standard of fabrication (by manual coating), the structure of the PS coating, and the RI of PS. The first factor is readily understood due to the uncertainties resulting from human skills. Meanwhile, it is noted that the geometry of the PS coating at the end of SMF influences the spectral response. For instance, PS coating with a parabolic shape did not produce a good spectral response, as shown in Figure S1 in supplemental materials, which might be because a large amount of light could be refracted. As a sensor, the flat PS-coated SMF demonstrated good responses via Fresnel reflection. The Fresnel reflection at the PS and air interface was much stronger than the reflection at the fiber and PS interface, which is further supported by Equation (3). An optical beam propagated from the SMF into the PS-polymer zone; consequently, the mode field expanded. The expanded beam was further reflected from the second interface, PS/air, and some of the reflected light was received by the acceptance core of the SMF. Therefore, the value of r_2_ was strongly related to the interference spectra.

PS is a thermosensitive polymer with diverse benefits, including versatility, simple fabrication, and inexpensive processing, and is thus considered as an excellent choice for thermo-optic sensors [42]. The thermosensitive properties of the fabricated sensors were characterized by changing the temperature. The Fresnel reflection optical intensity of the sensor head as a function of the temperature is represented in Figure S2 in supplemental materials. The Fresnel reflection optical intensity of bare SMF did not change considerably, whereas for the PS-coated sensor with the 8.0 μm thick layer, the Fresnel reflection optical intensity changed with respect to the temperature. This is attributed to the change in the RI and volume of the coated PS with temperature, which affects the optical intensity [17]. This data demonstrated a polynomial fit with R^2^ = 0.995, illustrating that this system is a good candidate as a fiber optic temperature sensor.

**Figure 5:**
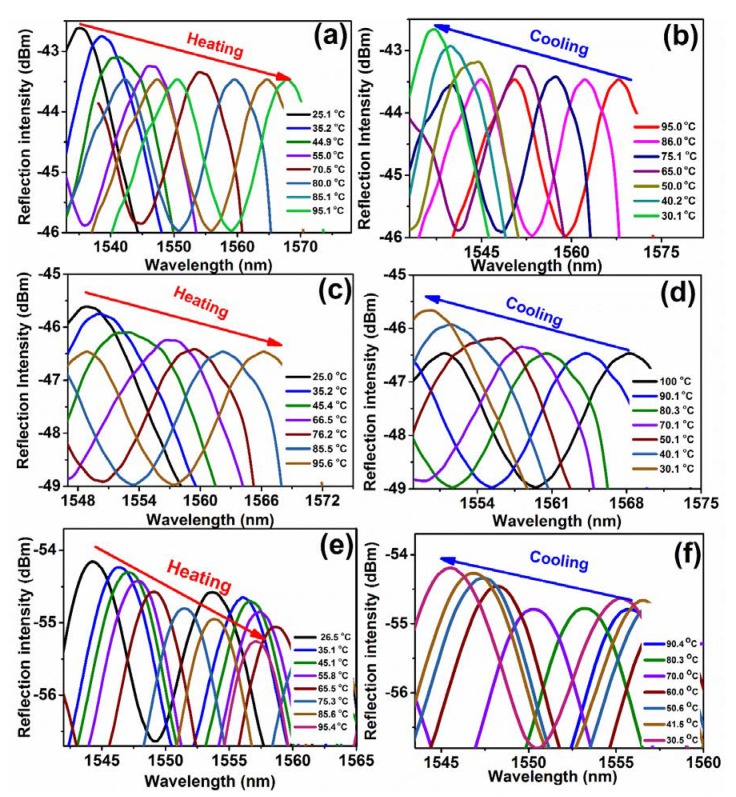
Reflection spectra of PS-coated SMF temperature sensors with a thickness of 2.0 μm (**a**,**b**), 4.1 μm (**c**,**d**), and 8.0 μm (**e**,**f**). A red shift in wavelength occurs with increasing temperature, while a blue shift in wavelength occurs with decreasing temperature.

The spectral response of the PS-coated sensor with temperature variation is shown in Figure S3 in supplemental materials. When the temperature was increased gradually from 25.0 to 100 °C with a step of 5 °C, the spectrum shifted to the longer wavelength direction (red shift), whereas the spectrum shifted toward the shorter wavelength direction (blue shift) with decreasing temperature. The simplified reflection spectra of the three sensors are depicted in Figure 5. The wavelength shifts result from the change the TOC and CTE of the polymer with temperature, which are responsible to the change of the optical path at the interface. Obviously the optical path determines the reflective spectrum from Equation (3) [43,44]. The changes in RI and coating thickness created a large optical path and led to a large phase difference at the interface.

**Figure 6:**
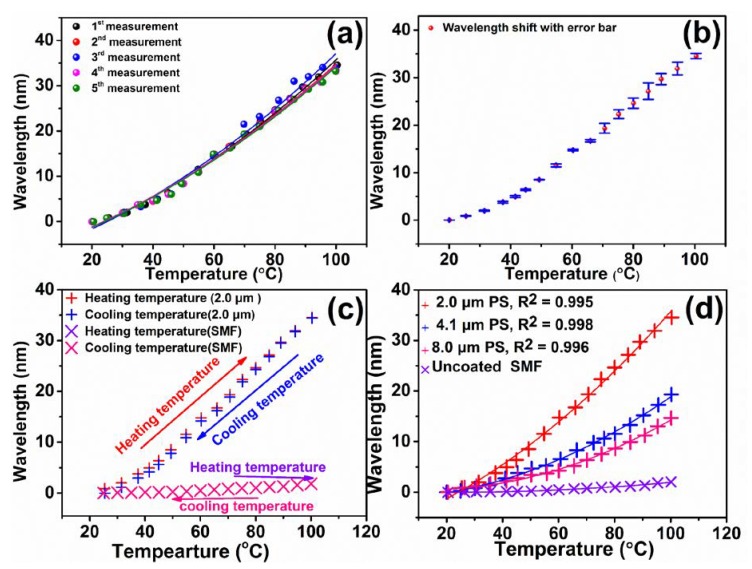
(**a**) Wavelength shift, (**b**) standard deviation for 2.0 μm PS coated sensor, (**c**) sensitivity of the proposed PS-coated (2.0 μm) SMF sensor and bare SMF, (**d**) sensitivity response of PS-coated SMF and bare SMF with difference in the thickness of the coating.

The five measured data of the sensor with PS coating (2.0 μm) is depicted in Figure 6a, and Figure 6b displays its standard deviation. In addition, Table 2 shows the standard deviations for the sensitivity of three different sensors. The measured standard deviations were 0.048, 0.042, and 0.03 pm °C^−1^ for the sensors with a thickness of 2.0, 4.1, and 8.0 μm coated with PS, respectively. These values indicate that the proposed sensors have good stability and reproducibility. The temperature response of the sensor (2.0 μm) was analyzed, and the corresponding wavelength shifts as a function of temperature in Figure S3a,b in supplemental materials are summarized in Figure 6c. The temperature response of bare SMF was not considerable. For the sensors with 2.0, 4.1, and 8.0 μm thick PS coatings, the average temperature sensitivity in the temperature range of 25–100 °C was 439.89, 219.39, and 147.52 pm °C^−1^, respectively. Figure 6d summarizes the results of the wavelength shift of three different sensors as a function of temperature from Figure S3a,c,e in supplemental materials. The fitting results were obtained by second-order polynomial fitting, which gave confidence factors of *R^2^* = 0.995, 0.998, and 0.996 for the sensors with 2.0, 4.1, and 8.0 μm thick PS, respectively, as shown in Figure 6d. This suggests that the sensor with 2.0 μm thin PS shows the highest sensitivity. When the thickness of coating is thin, the volume expansion could be less restricted as compared to that of a thick coating with respect to temperature. Consequently, the optical path could be notably changed for the sensor with thin coating, and results display a higher wavelength shift than that of the sensor with a thick coating of PS. The flat and thin coating is responsible for most of the r_2_ being recoupled to the core of the fiber [33,44,45]. Specifically, due to the introduction of PS polymer, which has high TOC and large RI in the 1550 nm wavelength window, the developed sensors could demonstrate high sensitivity, large dynamic range, and high temperature resolution. The sensor with thin PS coating (2.0 μm) creates the large FSR, which can provide the large dynamic range while the resolution is inevitably reduced due to the broadened fringes associated with the increased FSR. On the other hand, the dense fringes with small FSR for sensor with thick coating (8.0 μm) offers the high resolution of the temperature sensor. Nevertheless, the developed sensors with different coating thickness could achieve both the large dynamic range and the high temperature resolution [46,47].

Notably, the temperature-induced wavelength change of the sensor had better sensitivity at high temperature. Figure 6d proves that the spectral responses of such polymer-based Fabry–Perot interferometer sensors could be strongly associated with the temperature. The results demonstrate that the Fabry–Perot interferometer sensor had preferable sensitivity in the temperature region of 80–100 °C. For instance, the sensors with a 4.1 and 8.0 μm thick PS layer delivered sensitivities of 396.10 and 340.43 pm °C^−1^, respectively, in the high temperature region of 80–100 °C. The corresponding temperature sensitivity of the sensor with 2.0 μm thick PS was 510.28 pm °C^−1^, which is almost 51 times higher than that of the normal FBG temperature sensor, and 6.1 times higher than that of the long period fiber grating [21,33]. Consequently, it can be concluded that the proposed Fabry–Perot interferometer sensors could provide superior sensing performance via control of the PS coating thickness. A comparison of the characteristics of the fiber optic temperature sensors, operation temperature ranges, and temperature sensitivity are summarized in Table 3. The PS coated Fabry–Perot interferometer temperature sensor shows higher sensitivity than those of the Fabry–Perot interferometer with PVA, NOA-61, and PVC due to the considerably higher thermo-optic characteristics of the PS. It is noted that the sensitivities of the intrinsic Fabry–Perot interferometer are higher than those of extrinsic Fabry–Perot interferometer sensors [23,43,44]. However, the fabrication process of intrinsic Fabry–Perot interferometer sensors is complicated and requires expensive techniques. For instance, the air-microbubble PDMS based intrinsic Fabry–Perot fiber sensor requires a conventional fusion splicer (Fitel S178; Franklin, USA), mechanical fiber cleaver (Fitel S325, Franklin, USA), electric rail, and cleaving system for the fusion of the SMF and HCF [43]. Another example is the microfiber mode interferometer embedded in PDMS. The microfiber fabrication requires the modern glass processing machine (Vytran GPX -3400, Pittsfield, USA) for controlling the geometry and the dimensions of the fiber [23]. The fabrication of double polymer coated Fabry–Perot interferometer needs the step-curing ultraviolet photoresist (SU-8) technique for uniform and thin coating [44]. On the other hand, the extrinsic Fabry–Perot interferometer with a PS coating demonstrating high sensitivity was obtained by the simple dip coating method, which does not require expensive instruments and techniques. Most of the polymer coated sensors could be operated below 80 °C. Conversely, a PS coated temperature sensor could be operated up to 100 °C with good stability. The temperature sensitivity in a wide range achieved herein is much higher than previously reported values [21,33,42]. Moreover, the sensor with thick PS coating (8.0 μm) demonstrates higher temperature resolution than those of the sensors with PVA, NOA-61, PVC, and PC based on the values of FSR and TOC of the polymers [46,47]. Therefore, it can be considered that the proposed temperature sensor is suitable for highly precise temperature sensing.

**Figure 7:**
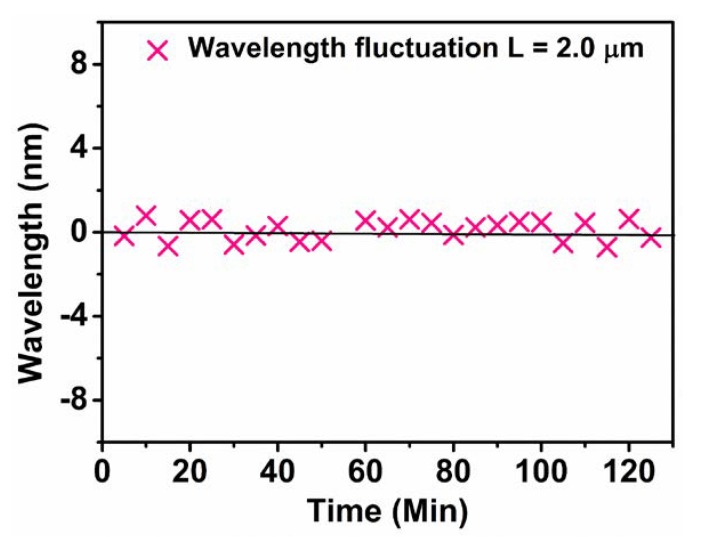
Wavelength fluctuation of the PS-coated (2.0 μm) sensor for 2 h.

Finally, in order to estimate the stability of the sensors, the wavelength of the resonant peak near 1550 nm was recorded as a function of time by maintaining the temperature at 25.0 °C for 2 h. Figure 7 shows the fluctuation of the wavelength for the PS-coated (2.0 μm) SMF sensor. The standard deviation of the wavelength and temperature over 2 h were about 0.19 nm and 0.09 °C, respectively, illustrating a high accuracy in fluctuation.

## 4. Conclusions

The Fabry–Perot interferometer temperature sensor based on thermosensitive PS-coated SMF was developed. The thickness of the sensing head was controlled by changing the solution concentration, which generated thicknesses of 2.0, 4.1, and 8.0 μm. The interference wavelength range and temperature sensitivity were largely dependent on the thickness of PS. The corresponding temperature sensitivities of the sensors were 439.89, 219.4, and 147.52 pm °C^−1^, respectively, in the temperature region of 25−100 °C. The sensitivity in different temperature ranges could be fitted by second-order polynomial fitting. Therefore, it is believed that these proposed sensors demonstrating high temperature sensitivity possess good potential for application to the measurement of the surrounding temperature for some physical and (bio) chemical sensing.

## Figures and Tables

**Table 1:** The properties of the different thermosensitive polymers.

Polymer	Refractive Index	TOC (°C^−1^)	CTE (°C^−1^)	Operational Temp (°C)	Ref.
Epoxy	1.438	−1.0 × 10^−4^	1.7 × 10^−4^	25–100	[38]
PMMA	1.48	−1.3 × 10^−4^	2.2 × 10^−4^	90	[31]
PC	1.585	−0.9 × 10^−4^	1.7 × 10^−4^	25–140	[31]
NOA 61	1.56 (1.541 before UV treatment)	−1.17 × 10^−4^	2.2 × 10^−4^	20–50	[33,39]
NOA 65	1.524 (1.515 before UV treatment)	−1.18 × 10^−4^	-	20–90	[40]
PVC	1.53 (1.546)	−1.14 × 10^−4^	2 × 10^−4^	20–60	[41,42]
PS	1.59	−1.2 × 10^−4^	2.2 × 10^−4^	20–100	This work

**Table 2:** Standard deviation of the sensitivity for three different PS sensors.

Sensor (PS Thickness)	Sensitivity (pm/°C)	Standard Deviation in Sensitivity (pm/°C)
2.0 μm	439.89	0.048
4.1 μm	219.39	0.042
8.0 μm	147.52	0.03

**Table 3:** Comparison of sensitivity of various fiber optic temperature sensors.

Types of Fiber	Polymer	T (°C)	Sensitivity (pm °C^−1^)	Ref.
Fabry–Perot Interferometer	PVA	25–100	~193.3	[21]
Fiber Bragg grating	NOA-61	10–50	19.5	[33]
Fiber Fizeau interferometer	NOA-61	10–50	269.5	[33]
Fabry–Perot Interferometer	PVC	25–60	366.0	[42]
Single mode + Hollow core fiber	HCF-PDMS	51–70.5	2703.5	[43]
Microfiber mode interferometer	PDMS	20–48	3101.7	[23]
Fabry–Perot Interferometer	Dual polymer capped PDMS	20–75	689.68	[44]
Fabry–Perot Interferometer	PC	20–140	245.4	[28]
Fabry–Perot Interferometer	PS	80–10025–100	510.28 439.89	This work

## Supplementary Materials

The following are available online at https://www.mdpi.com/1424-8220/20/3/794/s1, Figure S1: Optical microscopic image and spectral response of the parabolic shaped PS coated SMF; Figure S2: Reflection intensity change as a function of temperature; Figure S3: Detailed reflection spectra of PS coated SMF.
