# Supplementary material for: Enhancing Temperature Sensitivity of the Fabry–Perot Interferometer Sensor with Optimization of the Coating Thickness of Polystyrene
Source: Sensors (Basel). 2020 Jan 31;20(3):794. doi: 10.3390/s20030794 (PMC7038769; doi:10.3390/s20030794)
Supplement: Supplementary file 1 [file sensors-20-00794-s001.pdf]

Supporting Information

Enhancing Temperature Sensitivity of the Fabry-Perot Interferometer Sensor with Optimization of the Coating Thickness of Polystyrene

Tejaswi Tanaji Salunkhe[1], Dong Jun Lee[1], Ho Kyung Lee[1], Hyung Wook Choi[2], Sang Joon Park[1], Il Tae Kim,[1]*

[1] Department of Chemical and Biological Engineering, Gachon University, Seongnam-si, Gyeonggi-do 13120, Korea.

[2] Department of Electrical Engineering, Gachon University, Seongnam-si, Gyeonggi-do 13120, Korea

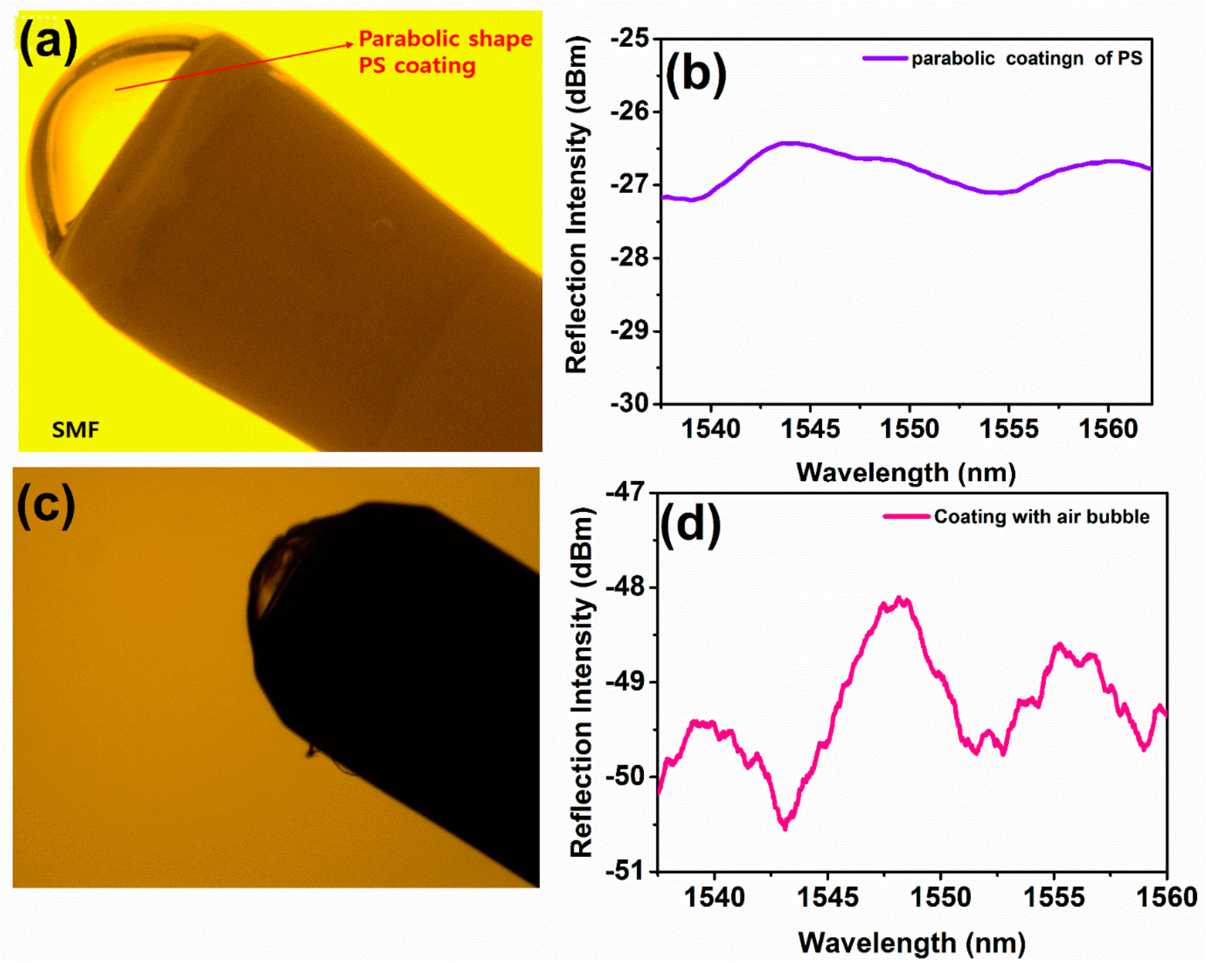

Figure S1. (**a**) Optical microscopic image of the parabolic shaped PS coated SMF. (**b**) Spectral response of the corresponding sensor. (**c**) Optical microscopic image and (d) the reflection spectra of the sensor containing air bubbles.

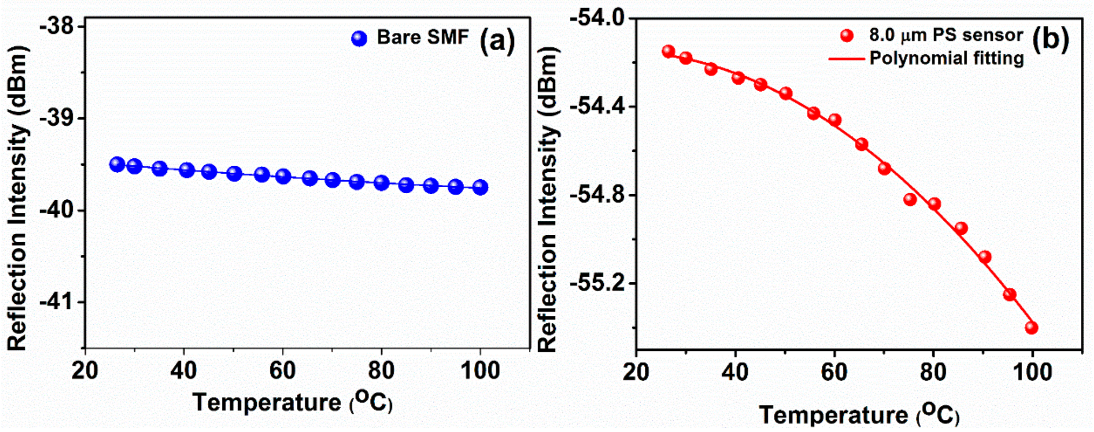

Figure S2. Change in reflection optical intensity of the sensor as a function of temperature. (**a**) Reference SMF and (**b**) PS coated sensor (8.0 μm).

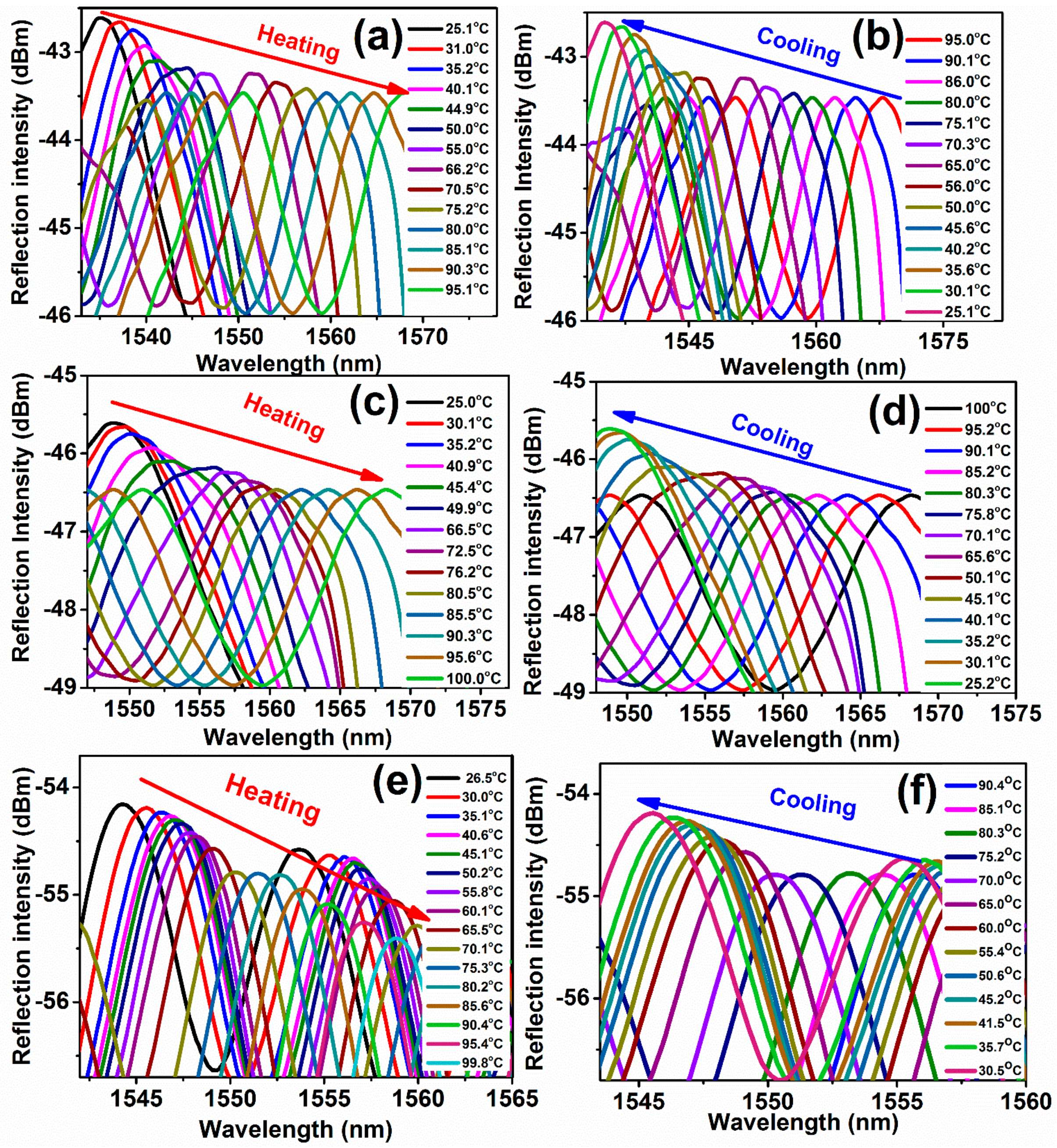

Figure S3. The reflection spectra of PS-coated SMF temperature sensors with a thickness of 2.0 μm (a-b), 4.1 μm (c-d) and 8.0 μm (e-f). A red shift in the wavelength occurs with increasing temperature, while a blue shift in the wavelength occurs with decreasing temperature. The reflection spectra were obtained by increasing the temperature in 5 °C intervals.
